# A Unified GNN-CV Framework for Intelligent Aerial Situational Awareness

**DOI:** 10.3390/s26010119

**Published:** 2025-12-24

**Authors:** Leyan Li, Rennong Yang, Anxin Guo, Zhenxing Zhang

**Affiliations:** Air Traffic Control and Navigation School, Air Force Engineering University, Xi’an 710051, China; li_leyan@163.com (L.L.); anxing0519@163.com (A.G.)

**Keywords:** command and control systems, computer vision, configuration recognition, intelligent situational awareness, swarm partitioning

## Abstract

Aerial situational awareness (SA) faces significant challenges due to inherent complexity involving large-scale dynamic entities and intricate spatio-temporal relationships. While deep learning advances SA for specific data modalities (static or time-series), existing approaches often lack the holistic, vision-centric perspective essential for human decision-making. To bridge this gap, we propose a unified GNN-CV framework for operational-level SA. This framework leverages mature computer vision (CV) architectures to intelligently process radar-map-like representations, addressing diverse SA tasks within a unified paradigm. Key innovations include methods for sparse entity attribute transformation graph neural networks (SET-GNNs), large-scale radar map reconstruction, integrated feature extraction, specialized two-stage pre-training, and adaptable downstream task networks. We rigorously evaluate the framework on critical operational-level tasks: aerial swarm partitioning and configuration recognition. The framework achieves an impressive end-to-end recognition accuracy exceeding 90.1%. Notably, in specialized tactical scenarios featuring small, large, and irregular flight intervals within formations, configuration recognition accuracy surpasses 85.0%. Even in the presence of significant position and heading disturbances, accuracy remains above 80.4%, with millisecond response cycles. Experimental results highlight the benefits of leveraging mature CV techniques such as image classification, object detection, and image generation, which enhance the efficacy, resilience, and coherence of intelligent situational awareness.

## 1. Introduction

Advances in distributed sensors, ultra-wideband communications, and high-performance computing have significantly boosted the speed, scale, and diversity of intelligence data collection in large-scale aerial operations. However, translating this unprecedented data deluge into actionable operational-level SA—a cornerstone for agile, data-driven decision-making in time-critical aerial command and control scenarios—remains a critical and pressing bottleneck. Traditional reliance on manual SA processes severely depletes human operators’ cognitive bandwidth, often leading to decision delays or suboptimal judgments in constrained operational cycles, which can directly jeopardize mission success in dynamic aerial confrontations [1].

This research focuses on advancing intelligent SA for aerial C2 systems, with a deliberate emphasis on the operational level, distinct from lower-level signal-layer analytics or spectral-optical imaging tasks. Core operational-level SA tasks include track prediction [2], swarm partitioning, object detection [3], threat assessment [4], and intent recognition [5,6], among others. A fundamental and consequential limitation of existing state-of-the-art approaches is their inherent fragmentation: these diverse SA tasks are typically addressed in isolation using specialized algorithms tailored to specific data structures (e.g., static snapshots, time-series trajectories) or individual task characteristics. This disjointed paradigm stands in stark contrast to the integrated, holistic decision-making process employed by human commanders who synthesize comprehensive radar map information to simultaneously infer multiple SA-related insights, highlighting a critical misalignment between computational methods and real-world operational needs.

Consider the exemplar challenge of swarm partitioning and configuration recognition. Current methodologies typically employ sequential, disparate algorithms: one for swarm partitioning [7], followed by another for configuration recognition [8]. This disjointed approach introduces cumulative computational latency and potential error propagation, compromising the accuracy and real-time efficiency essential for cooperative operational cognition. Crucially, human commanders overcome this fragmentation through end-to-end visual analysis of the radar map, directly inferring group divisions and formations holistically. This observation highlights a fundamental gap: the absence of a computational framework that emulates this efficient, vision-centric cognitive process for operational SA. We posit that a wide range of SA tasks can be effectively addressed within a unified, vision-centric deep learning framework processing the confrontation space as a radar-map-like image.

**Figure 1:**
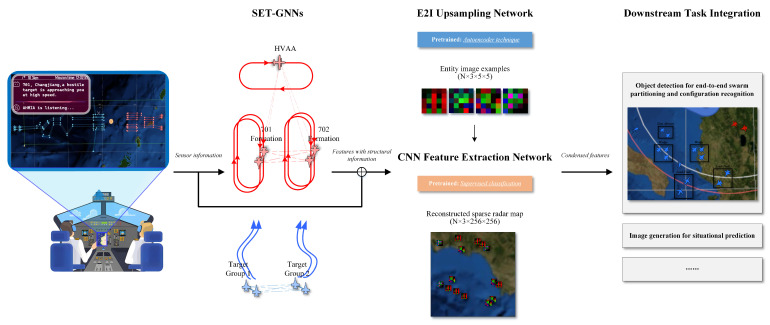
A unified GNN-CV framework for intelligent operational-level situational awareness.

Computer vision is a leading domain within artificial intelligence, incorporating robust models and techniques such as image classification [9,10], object detection [11,12], object tracking [13,14], and image generation [15]. However, applying computer vision directly to situation awareness encounters challenges. Firstly, the prevalent radar map is derived from secondary processing of static or temporal entity data within information systems, lacking a standardized and uniform image data structure. Additionally, the radar map exhibits sparse characteristics, with a large background and small entity targets. Consequently, commonly used image feature extraction networks and pre-trained weights obtained from datasets like ImageNet may not be entirely suitable for such sparse radar maps. Research utilizing Graph Neural Network (GNN) technology to reduce the sparsity of images has emerged as a widely studied research direction in the field [16,17]. Building upon these insights, we present a unified framework for addressing situational awareness challenges, covering the entire computer vision pipeline, as illustrated in Figure 1.

The framework includes:

- Sparse Entity Attributes Transformation Graph Neural Networks (SET-GNNs): Mitigates attribute sparsity within the image representation.
- Entity to Image (E2I) Upsampling Network: Reconstructs sparse radar images incorporating positional priors.
- CNN Feature Extraction Network: Demonstrated as effective for sparse radar imagery theoretically and empirically.
- Two-Stage Pre-training Method: Enables training of specialized image feature extractors.
- Downstream Task Integration: Extends the framework to diverse SA tasks (e.g., trajectory prediction, situation assessment).

It is critical to note that our framework operates on outputs from integrated C2 information systems. These systems fuse data from diverse radar/intelligence sources (e.g., Doppler radar providing velocity/azimuth, lidar providing range/3D shape) into a standardized input representation (e.g., target latitude, longitude, speed, altitude, radar cross-section). We empirically validate the framework’s efficacy and efficiency using two core SA tasks: aerial swarm partitioning and configuration recognition. The paper is structured as follows: Section 2 covers related work; Section 3 details the proposed model framework; Section 4 presents experimental results for the two tasks; Section 5 concludes the work and outlines future directions.

## 2. Related Work

### 2.1. Aerial Swarm Partitioning

Aerial swarm partitioning refers to the segmentation of collective aerial targets into cohesive and interacting subgroups, typically leveraging hierarchical structures and spatial relationships. Significant research efforts [18,19,20,21] have focused on enhancing the clustering of target groups, prioritizing improvements in accuracy, stability, and responsiveness during dynamic force aggregation scenarios. These approaches predominantly build upon advanced clustering algorithms, often augmented with domain-specific knowledge. For instance, Yan et al. [18] proposed a cell-based density clustering algorithm effective for grouping large-scale aircraft formations, particularly when the cluster count is unknown a priori. Ma et al. [19] enhanced the density peak clustering framework by incorporating manifold distance metrics and adaptive cluster center selection, enabling automated grouping of aerial targets.

### 2.2. Formation Configuration Recognition

Current methodologies for aerial formation recognition can be broadly categorized into two paradigms:

- Template Matching: This approach utilizes domain expertise to define preset formation templates, matching them against observed targets based on spatial relationships. Zhang et al. [22] combined Hough transform with clustering to identify naval formations. Leng et al. [23] leveraged domain knowledge to recognize formations by detecting key structural elements like reference targets and queue lines. While interpretable, these rule-based methods lack flexibility and exhibit limited robustness against noise, occlusions, or significant deviations from templates.
- Computer Vision: This paradigm constructs abstract graphical representations of formations and employs deep learning models, primarily CNNs, for classification [8,24,25,26,27]. Studies such as [8,24] encode target positions using binary (0,1) matrices as input to image classification networks. A critical limitation of this binary encoding is the omission of essential kinematic attributes (e.g., heading, velocity), constraining algorithm versatility and reducing recognition accuracy, especially for complex motion-based formations.

## 3. Methodology

This section presents the components of the proposed GNN-CV architecture for intelligent operational-level SA. Firstly, we introduce a sparse map reconstruction method using SET-GNNs with an E2I upsampling network and a CNN feature extractor. Subsequently, we develop a two-stage pre-training strategy to enable rapid adaptation of the pre-trained networks to downstream tasks. Finally, we apply an end-to-end object detection algorithm for aerial swarm partitioning and configuration recognition problems, effectively validating the rationale of the theory.

### 3.1. Sparse Entity Attribute Transformation Graph Neural Networks

Entity attributes for large-scale situation images are often independent and sparse, making it difficult to reconstruct the overall image. To address this issue, we aim to incorporate structural information into entity features before transforming them into images. GNNs are a popular choice for modeling graph structure information through message passing mechanisms, enabling the reduction of sparsity in entity attributes.

Basic GNN theory is not discussed in depth in this paper. Commonly used GNN models include GCN [28], GAT [29], GraphSAGE [30], GIN [31], and their variants, each with unique characteristics that affect problem-solving efficacy. The formula representation of the current mainstream graph neural network is given below.

(1) GCN:     hv(l+1)=σW(l)∑u∈N(v)1cuvhu(l)(2) GAT:     euv=LeakyReLUAttention(hu(l),hv(l))(3)       auv=exp(euv)∑k∈N(v)exp(evk)(4)       hv(l+1)=σ∑u∈N(v)auv·W(l)hu(l)(5) GIN:     hv(l+1)=MLP(1+ϵ)·hv(l)+∑u∈N(v)hu(l)

Applying GNNs directly to entity data feature transformation encounters an obstacle in the absence of a natural adjacency matrix to represent graph connections. To address this, we propose three graph adjacency matrix construction methods. The first method utilizes an all-ones matrix, indicating that all entities are related with each other and have an edge weight of 1. Building on this, the second method determines edge weight based on the location difference of entities, where the weight is inversely proportional to the relative distance between nodes. The third method constructs adjacency matrix according to the relative distance between nodes and a group distance threshold relationship. The formula representation of these methods is provided below.

(6) Method 1:     g1[i,j]=1(7) Method 2:     g2[i,j]=1−D(i,j)dmax(8) Method 3:     g3[i,j]=1,if D(i,j)≤dthreshold0,otherwise

where $g_{1}$, $g_{2}$, and $g_{3}$ denote three adjacency matrices, D(i,j) denotes the relative distance between entities, $d_{\max}$ denotes the maximum possible entity distance, and $d_{\mathrm{threshold}}$ denotes as the designated distance threshold for groups. These methods are designed based on the characteristics of the situation awareness problem domain.

### 3.2. E2I Upsampling Network and Matched Feature Extraction Network

The E2I network utilizes deep learning to convert structural features into depth images, albeit not directly into standard sizes like 256×256 or 420×420. Initially, the E2I network translates structural features extracted from GNNs into depth images of size (n×n), integrating this information into a radar situational map based on latitude and longitude positions (256×256/420×420). This reconstruction method leverages the strength of convolutional networks in processing location data. Empirical evidence underscores the benefits of incorporating prior positional details. It is crucial to highlight that reconstructed radar situational maps diverge from traditional images (e.g., photos, video frames) due to the absence of graphical edges, color data, and the presence of relatively small entities in a vast situational expanse, resulting in distinct sparsity within the radar situational maps.

**Figure 2:**
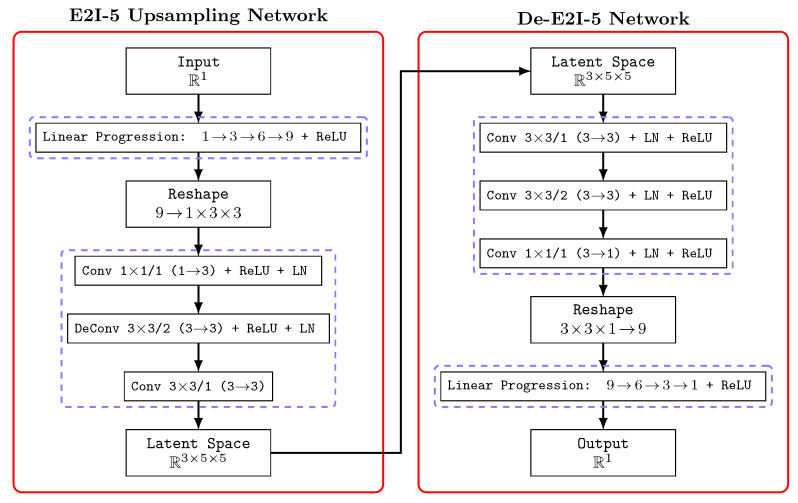
E2I-5 upsampling network and its decoder (De-E2I-5), which is used in the pre-training phase.

The design of the E2I upsampling network requires careful consideration of its interaction with the feature extraction network. Mainstream image feature extraction networks typically employ a pyramid convolutional structure, consisting mainly of multiple 3×3 convolutional layers and residual connections. Therefore, our E2I network adopts a square-shaped design with dimensions of n×n, which aligns with standard computer vision methodologies and maintains the entity images as three-channel outputs. For example, when n=5 and there is one entity attribute present, the structure of the E2I-5 network is detailed in Figure 2.

The E2I network incrementally processes entity attributes to a 3×n×n format. Drawing inspiration from convolutional module designs found in networks like ResNet [32] and AlexNet [33], the E2I network incorporates multiple ReLU activation layers to enhance non-linear fitting capacity, along with LN layers to expedite network training speed.

**Theorem** **1.** *Traditional shallow convolutional neural networks, comprising 3×3 convolution kernels, demonstrate proficiency in extracting small objects and non-edge features.*

The theorem proof is provided in Appendix A. The reconstructed radar situational map introduced in this research exhibits sparse, edgeless, and structured attributes. In addition, not all established feature extraction networks like ResNet50, AlexNet, DenseNet [34], etc., are represented. Consequently, the shallower layers of ResNet18 demonstrate higher efficiency in feature extraction, as supported by the experimental Section 4.2.1. For further clarification, Table 1 outlines the subnetwork structure for feature extraction within ResNet18.

### 3.3. Pretrained Tasks

The reconstructed radar map and feature extraction network can benefit from pre-training techniques for weight initialization, thereby reducing the fine-tuning costs for subsequent tasks. In this study, the sparse radar map reconstruction involves the E2I and ResNet18 feature extraction networks. To address this challenge, we develop a two-stage pre-training process outlined in Figure 1.

The initial step involves unsupervised pre-training of E2I using the autoencoder technique [35]. This ensures that the resulting three-channel n×n images effectively capture the transformed entity attributes, aligning the encoding and decoding processes accordingly. We utilize the E2I-5 network described in Section 3.2 as the encoder and design a symmetric decoder to reconstruct the upsampled entity images back to their original attribute outcomes. The De-E2I-5 network structure is detailed in Figure 2.

After pre-training E2I network, we freeze it and proceed to pre-train the feature extraction network. Common methods for pre-training image feature networks include Masked Autoencoder [36] and unsupervised contrastive learning. However, Masked Autoencoder, which predicts masked pixels in images, is not suitable for sparse radar image reconstruction in our study. Instead, we opt for unsupervised contrastive learning [37] but utilize supervised learning for feature extraction through a classification method. By creating labeled flight formation radar state maps, we employ supervised image classification for pre-training. We adjust only the output categories of the final layer’s classification head to six types of flight formation configurations on ResNet18, successfully completing the pre-training of the ResNet18 feature extraction network in conjunction with the E2I network.

### 3.4. Object Detection Method for End-to-End Swarm Partitioning and Configuration Recognition

The technology for end-to-end swarm partitioning and configuration recognition involves three primary steps: swarm database construction, radar map reconstruction, and implementation of the object detection algorithm.

In the absence of labeled swarm databases, we propose a method to generate uniformly distributed configuration data based on six typical flight formation configurations. The key parameters and features of these configurations are outlined in Appendix B. Our study underscores the importance of positional relationships and aircraft headings in assessing formation configurations based on radar map observations. Therefore, our method generates configuration data by reverse-engineering the positions and headings of individual aircraft within the formation according to the formation type. To enhance database diversity, we incorporate substantial randomization and noise. The algorithm produces data in a list format containing the horizontal and vertical coordinates, along with the headings of each aircraft. The pseudocode Algorithm 1 illustrating the method for generating flight formation configuration data is provided.
**Algorithm 1** Formation Configuration Data Generation1:**procedure** GenerateData(category)2: **if**
category∈ {Lead-Trail, Line, Swept} **then**3:  numAircraft←rand(2,4)4: **else if**
category∈ {Wedge, Stinger} **then**5:  numAircraft←36: **else if**
category∈ {Container} **then**7:  numAircraft←48: **end if**9: courses←randomCourses()10: formationList←∅11: **while**
|formationList|<numAircraft
**do**12:  **if**
formationList=∅
**then**13:   leaderCoords←randomInitCoords()14:   formationList←{leaderCoords}15:  **end if**16:  dist←randomDist(configCategory)17:  newCoords←generateCoords(dist,angels)18:  **if**
newCoords∉[0,255]
**then**19:   formationList←∅20:  **else**21:   add newCoords to formationList22:  **end if**23: **end while**24: noisyFormation←addNoise(formationList)25: **return**
noisyFormation26:**end procedure**

Notably, experimental findings in Section 4.3 demonstrate that the DETR method achieves the highest accuracy for end-to-end recognition. DETR utilizes a CNN backbone for initial image feature extraction and compression. These compressed features undergo flattening, positional encoding augmentation, and are input into a transformer. We propose that DETR leverages the integration of feature compression and the attention mechanism to demonstrate robust sparse image object detection capabilities.

## 4. Experiment

### 4.1. Sparse Attributes Transformation Graph Neural Network

The introduction of the GNN architecture represents a crucial innovation in the model for extracting entity structural attributes from situation information. In this section, we perform experiments on the construction of adjacency matrices and the GNN model architectures.

#### 4.1.1. Adjacency Matrix Construction Method

To apply GNN for extracting structural information, an adjacency matrix needs to be constructed between the entities of situation information. This study experimentally compares three construction methods outlined in Equations (6)–(8) in Section 3.1 to determine the most optimal adjacency matrix. A two-layer GCN model with a hidden layer dimension of 32 is utilized, along with a ReLU activation layer following each layer. The experiments involve using E2I-5, ResNet18, and DETR for swarm partitioning and configuration recognition in downstream networks. Training is conducted for 20 epochs with 50 iterations per epoch, starting with an initial learning rate of $10^{-2}$ and decay by 0.1 every 10 epochs. Specifically, $d_{\max}$ in Equation (7) is set at 300 and $d_{\mathrm{threshold}}$ in Equation (8) is set at 15. The results of the experiments are summarized in Table 2.

The experimental results demonstrate that Method 2 utilizes entity relative distance to adjust the weight of adjacency matrix, aligning with the problem scenario of situation cognition and offering more structural information. This method is further employed in subsequent experiments for adjacency matrix construction.

#### 4.1.2. Ablation

To identify the optimal GNN model architecture, this section employs variable control methods and conducts a series of ablation experiments on GNNs. The basic experimental setup is consistent with Section 4.1.1.

- w/o GNNs: We remove the GNNs part of the model and directly deal with sparse entity features with E2I network.
- w/o residual connection-1: We remove the residual connections before and after GNN in the model.
- w/o residual connection-2: We change the residual connection before and after GNN from summation to concatenation.

The results in Table 3 illustrate the outcomes of a series of ablation experiments. The experimental findings indicate that incorporating a GNN model for extracting structural features of sparse entities can alleviate the sparsity characteristics of situation maps to a certain degree. Among the GNN variants (GCN/GIN/GAT) in this model, GCN demonstrates superior performance. Furthermore, integrating residual connections into the GNN model enhances its overall performance.

### 4.2. E2I Upsampling Network and Matched Feature Extraction Network

The size and structure of the E2I upsampling network, as determined by Theorem 1, significantly impact the effectiveness of radar map reconstruction in downstream applications. Through experiments, we identify the optimal image size and corresponding feature extraction network for improved radar map reconstruction.

#### 4.2.1. Image Classification for Matching Image Features and Extraction Networks

In image classification tasks, the network comprises the input image, backbone feature extractor, and classification head. This setup enables a direct evaluation of the synergy between image reconstruction and feature extraction networks. To determine the ideal size for the E2I-n network and its paired feature extractor, we conducted configuration classification experiments using one flight formation per radar map. Our experiments assessed the compatibility between entity images at varying scales and conventional image feature extraction networks like ResNet and AlexNet. The initial learning rates were set at $1\times 10^{-4}$ for ResNet18, $2\times 10^{-5}$ for ResNet50 and ConvNeXt-Tiny, and $5\times 10^{-5}$ for ResNet101 and AlexNet. These rates decay by 0.1 every 10 epochs during the 40-epoch training process, with 100 iterations per epoch and a batch size of 64, using CrossEntropy loss. Specific experimental findings are detailed in Table 4.

The experiments underscore the superior performance achieved by pairing the E2I-5 upsampling network with ResNet18 for feature extraction, surpassing other network combinations. Moreover, the convergence speed of the E2I-5 and ResNet18 combination significantly outpaces that of other combinations. Employing ResNet18, a compact backbone network, the feature extractor effectively complements the sparse radar reconstruction images. These findings support the conclusions drawn from Theorem 1 at an experimental level. Furthermore, we visualized layer activations from ResNet18/50/101 to analyze shallow network performance, demonstrating comparable capability to deeper networks on sparse images. Visualizations are provided in Appendix C.

#### 4.2.2. Ablation

After conducting the configuration classification experiment outlined in Section 4.2.1, a series of ablation experiments were performed to validate the efficacy of the proposed E2I upsampling network architecture:

- w/o LN: We remove LN layers in the E2I network.
- w/o RELU: We remove ReLU activation in the E2I network.
- w/o Linear-2: We remove the second Linear layer and make adjustments to the input–output dimensions of linear Layers 1 and 3 in the E2I network.
- w/o prior location information: Rather than relying on prior position information, we directly upsample all flight attributes to a standard image size of 3×256×256.

**Figure 3:**
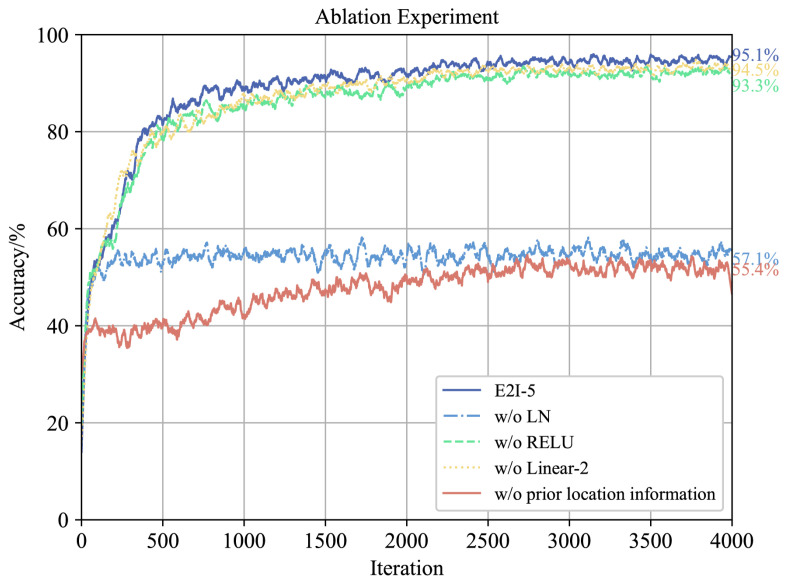
Results of ablation experiments for the E2I-5 network.

Refer to Figure 3 for the convergence results of classification accuracy during training across various cases. The experimental findings demonstrate the effectiveness of the developed E2I network structure and sparse radar map reconstruction method.

### 4.3. End-to-End Swarm Partitioning and Configuration Recognition

In this section, we first compare the recognition accuracy of three prominent object detection algorithms. Subsequently, we conduct three sets of robustness tests tailored for aerial application scenarios. Lastly, we demonstrate the practical application effect.

#### 4.3.1. Comparison of Classical Object Detection Algorithm in the Method Based on Reconstruction Sparse Radar Map

We assess the end-to-end recognition accuracy of three object detection architectures: Faster R-CNN, YOLOv5, and DETR. The experiment utilizes the E2I-5 upsampling network and ResNet18 backbone network, pre-trained as described in Section 3.3. Details concerning the pre-training of the image feature extraction network and the training process of the overall model are provided in Appendix D and Appendix E. The recognition accuracy results for the three object detection algorithms are summarized in Table 5.

Based on the results presented in Table 5, it is evident that ResNet18, used as the backbone network, significantly outperforms larger backbone networks. This difference in performance could be attributed to suboptimal training parameters, challenges associated with training larger networks, and other contributing factors. However, it is suggested that larger networks excel in capturing intricate edge and detail features within images, although this might not align well with the scale of formation configuration images. On the other hand, the ResNet18 network, focusing on image scale, aligns better with formation configuration images, aiding in the recognition of formation configurations through depth of field analysis and aircraft positioning relationships. Furthermore, DETR, a computer vision network leveraging transformer architecture, outperforms pure CNN architectures due to the transformer’s self-attention mechanism, which excels at capturing sparse features within images.

**Figure 4:**
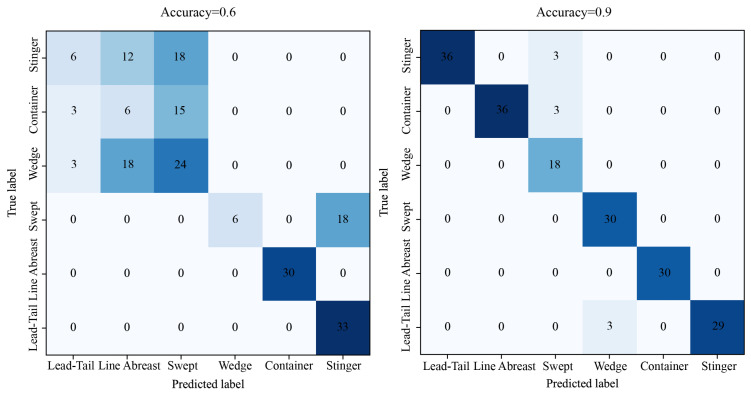
Confusion matrix for end-to-end swarm partitioning and formation configuration recognition.

The confusion matrix illustrates the outcomes of configuration classification during the training phases of the DETR network, achieving accuracies of approximately 0.6 and 0.9, as depicted in Figure 4. Analysis of the sparse matrix reveals that at 0.6 accuracy, DETR demonstrates proficient anchor box regression and basic shape recognition capabilities, distinguishing between Lead-Trail/Line Abreast/Swept, Container, and Wedge/Stinger formations, yet it lacks the ability to capture heading information and depth of field representation in images. With further training, at an accuracy of 0.9, only a subset of configurations is misclassified, highlighting the seamless integration of the E2I network with DETR’s overarching architecture.

#### 4.3.2. Robustness Analysis of End-to-End Partitioning and Configuration Recognition in the Method Based on Reconstruction Sparse Radar Map

In real-world aerial confrontation scenarios, numerous sources of information interference and uncertainty are present. To evaluate the robustness and practical applicability of computer vision technology in downstream tasks utilizing the sparse radar map reconstruction method, this section examines two factors potentially impacting recognition outcomes for end-to-end partitioning and configuration recognition: information noise errors and aircraft spacing within formations.

Firstly, we assess the algorithm’s robustness under significant levels of information noise interference. Information noise errors primarily stem from two sources: radar detection inaccuracies due to detection errors and enemy interference as well as operational deviations resulting from the flight control process. To guarantee the resilience of the algorithm and its efficacy in practical applications, this section constructs a formation recognition database utilizing noise indicators outlined in Table A1. These indicators encompass positional deviations within ±1 km and heading deviations within ±11.5°, categorized as 1× noise. Supplementary positional and heading noise factors are incorporated to evaluate the precision of formation recognition. The algorithm’s capacity to withstand noise interference is demonstrated in Table 6.

**Figure 5:**
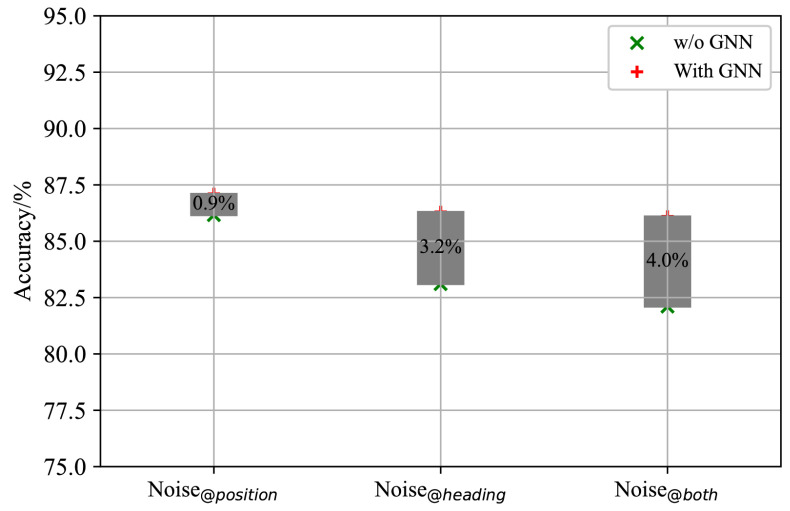
Effects of the GNN module on enhancing the model’s anti-noise ability.

Due to the unique message-passing mechanism of GNNs, aircraft in formation exhibit “homogeneity,” similar to complex averaging through high-dimensional operations. Based on this, we hypothesize that incorporating GNNs improves the model’s resistance to interference, which we test through experiments. In these experiments, training parameters are kept constant. The results in Figure 5 show that course noise has a more significant impact on identification accuracy than coordinate noise. The GNN module notably enhances the model’s resilience to course noise when entity attributes are fused. However, the model’s performance against coordinate noise appears to be influenced primarily by the convolutional neural networks.

Secondly, we assess the accuracy of end-to-end partitioning and configuration recognition under diverse flight intervals. In formation flying, the spacing between aircraft is tactically adjusted based on mission objectives and aircraft capabilities. During covert operations, aircraft fly in close formation to reduce exposure risks, creating the illusion of a larger entity. Conversely, in routine training exercises, a more conservative spacing is adopted for safety. However, this variability poses a challenge for computer vision techniques. The relationship between recognition accuracy and flight interval is illustrated in Table 7.

Table 7 presents the recognition accuracy of fixed flying intervals within formations. The algorithm exhibits strong versatility, achieving high accuracy even when the intervals between aircraft within the same formation are not uniform. While there may be a reduction in accuracy with excessively small or large formation intervals, this phenomenon is directly linked to the model range specified in Table A1 for the configuration recognition database. As indicated in Table A1, the model’s flying intervals are constrained within the range of [5 km, 15 km], rendering cases such as 3 km and 20 km as novel formation image types for the algorithm. This study aims to expand the flying intervals in the formation configuration model to a broader range of [1 km, 20 km] during network training. This adjustment is anticipated to enhance the recognition accuracy of formations with smaller or larger (1 km/25 km) flying intervals by 5–10%; however, the overall average recognition accuracy is projected to decrease to 87.9%.

#### 4.3.3. Practical Application Effect of End-to-End Swarm Partitioning and Configuration Recognition Algorithm

**Figure 6:**
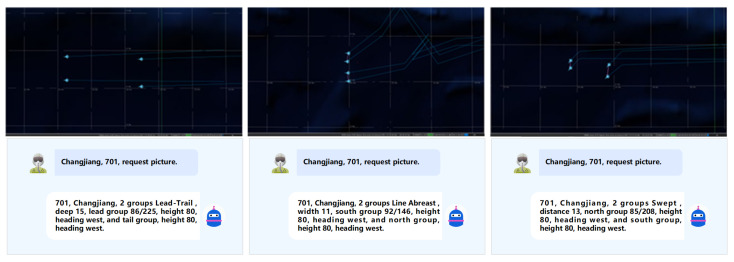
Application effects of the proposed end-to-end algorithm.

To evaluate the effectiveness of the proposed GNN-CV architecture in realistic air command and control systems, we deployed a representative air confrontation scenario on a human–machine interaction (HMI) simulation platform. The proposed end-to-end swarm partitioning and configuration recognition algorithm is initiated by the “request picture” voice command, which triggers the tactical display generation process. The swarm partitioning and configuration recognition results for the aerial formations are presented in Figure 6.

Validation results demonstrate the efficacy of integrating graph neural networks and computer vision techniques with radar map reconstruction. Experimental evaluations conducted on the i9-12900K CPU and NVIDIA A5000 GPU platform reveal that the target detection framework, which employs a six-layer eight-head DETR architecture with ResNet18 as the backbone, yields an approximate throughput of 30 FPS and exhibits a millisecond-level recognition latency. Crucially, in engineering practice, the flight separation distance can be readily scaled to the model’s input range via straightforward multiplicative or divisive operations, without compromising identification accuracy. This approach has undergone rigorous real-world validation, yielding robust performance outcomes.

## 5. Conclusions

This paper introduces a unified framework that leverages computer vision techniques to address various situational awareness challenges. We propose SET-GNNs and E2I upsampling network that utilize prior location data to convert sparse entities into standard image formats. To efficiently extract features from reconstructed radar images, we advocate for shallow pyramid structure convolutional neural networks, demonstrating their efficacy through both theoretical analysis and practical application. To facilitate the integration of image conversion and feature extraction networks across diverse situational awareness tasks, we introduce a two-stage pre-training approach for the relevant networks. We validate our framework through aerial swarm partitioning and configuration recognition tasks. The experimental results confirm the architecture’s utility, effectiveness, and resilience, aligning with human cognitive principles.

The provision of databases tailored for SA training in computer vision is likely to be the primary obstacle that restricts the further application of this model. In the future, we anticipate leveraging this architecture to explore applications such as target tracking and video generation for situational prediction. Additionally, we aim to employ image segmentation techniques to achieve large-scale situational awareness.

## Figures and Tables

**Table 1:** ResNet18 network structure.

Layer	Size	Output
Conv module 1	7 × 7, 64	112 × 112
Conv module 2	3 × 3 Max pool $\left[\begin{array}{c} 3\times 3,64\\ 3\times 3,64 \end{array}\right]\times 2$	56 × 56
Conv module 3	$\left[\begin{array}{c} 3\times 3,128\\ 3\times 3,128 \end{array}\right]\times 2$	28 × 28
Conv module 4	$\left[\begin{array}{c} 3\times 3,256\\ 3\times 3,256 \end{array}\right]\times 2$	14 × 14
Conv module 5	$\left[\begin{array}{c} 3\times 3,512\\ 3\times 3,512 \end{array}\right]\times 2$	7 × 7

**Table 2:** Experimental results for different adjacency matrix forms.

Adj. Matrix	Meth. 1	Meth. 2	Meth. 3
Accuracy	77.7%	84.2%	78.5%

**Table 3:** Different GNN architectures and related ablation experimental results.

Setup	GNN Architectures			Ablation Studies		
	GCN	GIN	GAT	w/o GNNs	w/o res.1	w/o res.2
Accuracy	84.2%	83.6%	83.8%	80.5%	79.8%	83.4%

**Table 4:** Matching relationship between E2I-n and multiple feature extraction networks.

Backbone	Params	E2I-3_@ACC_	E2I-5_@ACC_	E2I-7_@ACC_	E2I-17_@ACC_
ResNet18	11.2M	92.0%	**95.1%**	92.3%	57.0%
ResNet50	23.5M	87.7%	92.1%	89.6%	78.5%
ResNet101	42.5M	94.4%	95.0%	93.3%	87.0%
AlexNet	57.0M	65.7%	54.4%	54.5%	53.7%
ConvNeXt	27.8M	67.8%	72.6%	56.6%	55.1%

*Note:* The top **1^st^** is highlighted.

**Table 5:** Different object detection algorithms for end-to-end partitioning and configuration recognition.

Achitecture	Backbone	Params	Pre-Trained	Accuracy
Faster-RCNN	ResNet18	28M	w/o pretrained	49.8%
			ImageNet pretrained	50.1%
			special pretrained	62.3%
	ResNet50	41M	ImageNet pretrained	50.0%
	ResNet101	60M	ImageNet pretrained	50.1%
YOLO	v5s	7M	w/o pretrained	60.0%
	v5m	21M	w/o pretrained	61.2%
	v5l	46M	w/o pretrained	57.5%
DETR	ResNet18	29M	w/o pretrained	84.2%
			ImageNet pretrained	86.1%
			special pretrained	**90.1%**
	ResNet50	41M	ImageNet pretrained	83.7%
	ResNet101	60M	ImageNet pretrained	85.4%

*Note:* The top **1^st^** is highlighted.

**Table 6:** Anti-noise ability of the algorithm.

Range of Noise_@1×_	Extra Noise Ratio	Accuracy
Coord. (km): [−1, 1] Course (rad): [−0.2, 0.2]	0	90.1%
	0.5	87.2%
	1	86.1%
	2	80.4%

**Table 7:** End-to-end recognition accuracy of different flight intervals.

Flight Interval_@train_	Flight Interval_@test_	Accuracy
[5 km, 15 km]	0 km	90.1%
	3 km	85.0%
	5 km	87.0%
	8 km	89.2%
	10 km	89.8%
	12 km	89.9%
	15 km	89.8%
	20 km	87.3%

**Figure A1:**
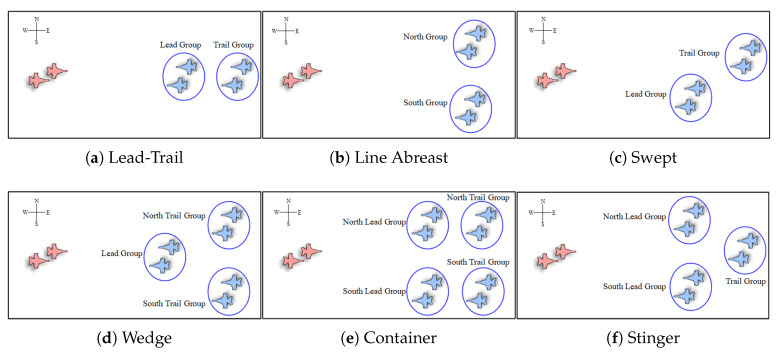
Typical flight formation.

**Figure A2:**
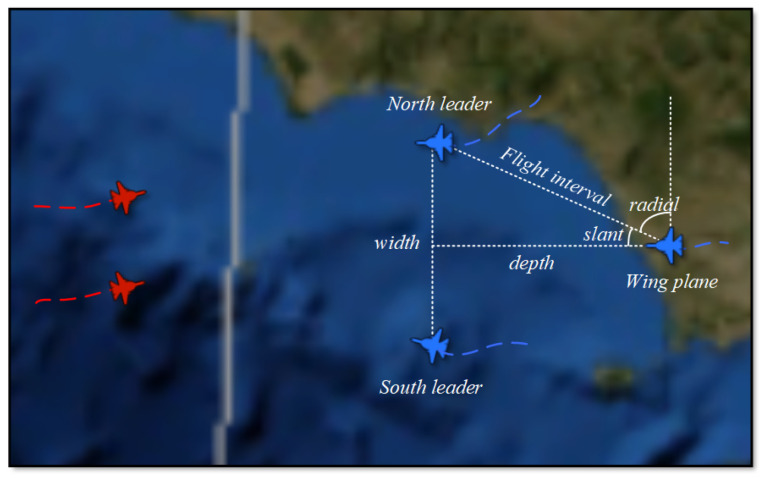
Key parameters of the Stinger formation.

**Figure A3:**
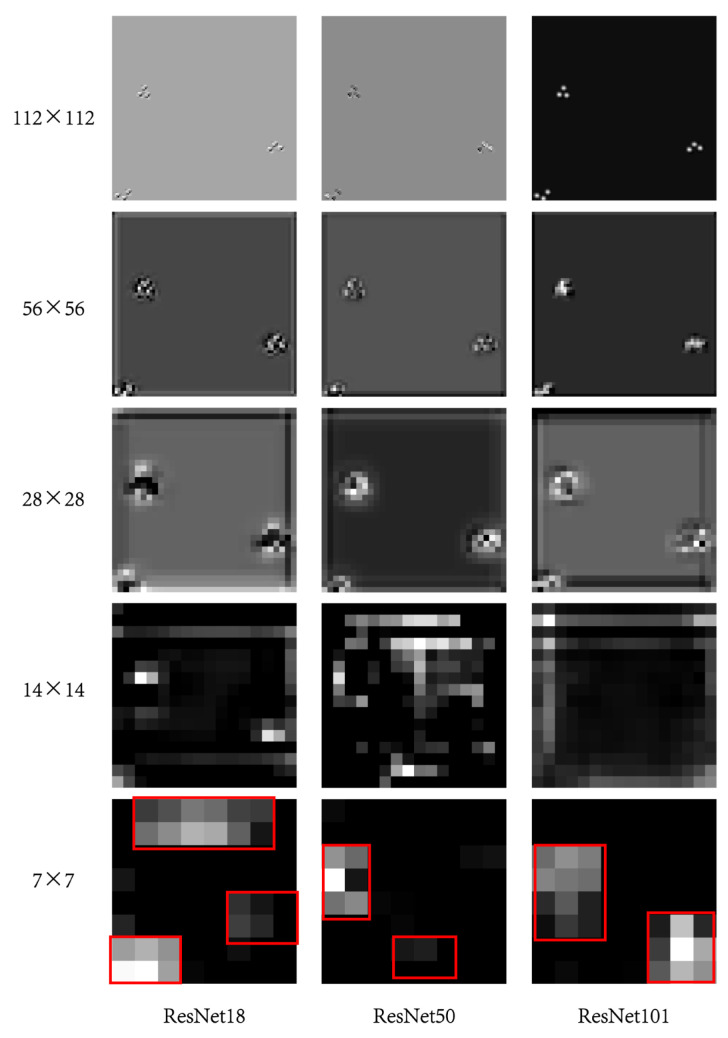
Visualization of the feature extraction layers (ImageNet pretrained). The red boxes highlight the significant image features extracted by different feature extraction networks.

**Figure A4:**
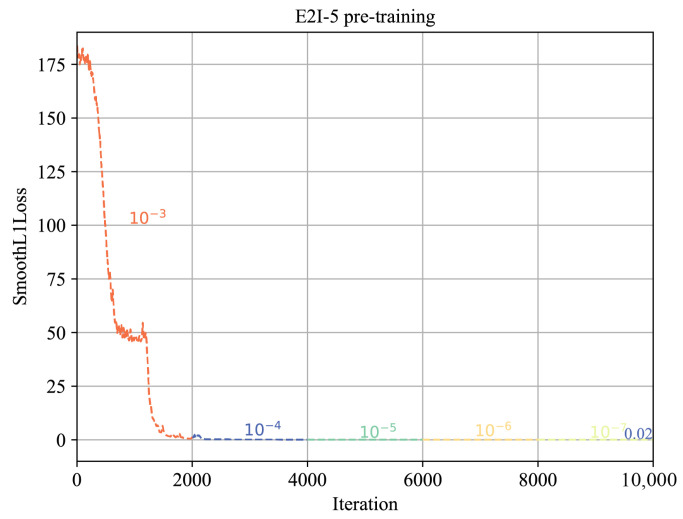
The pre-training process for E2I-5. Different colors are employed to denote distinct learning rates.

**Figure A5:**
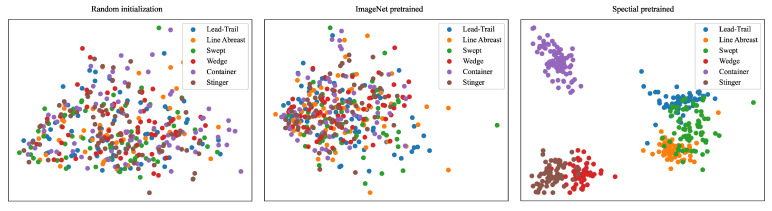
The 2D projections of extracted representations through PCA. The feature extraction outcomes are from three networks: one with randomly initialized weights, another pre-trained on ImageNet, and the specific pre-trained image feature extraction network employed in this study.

**Figure A6:**
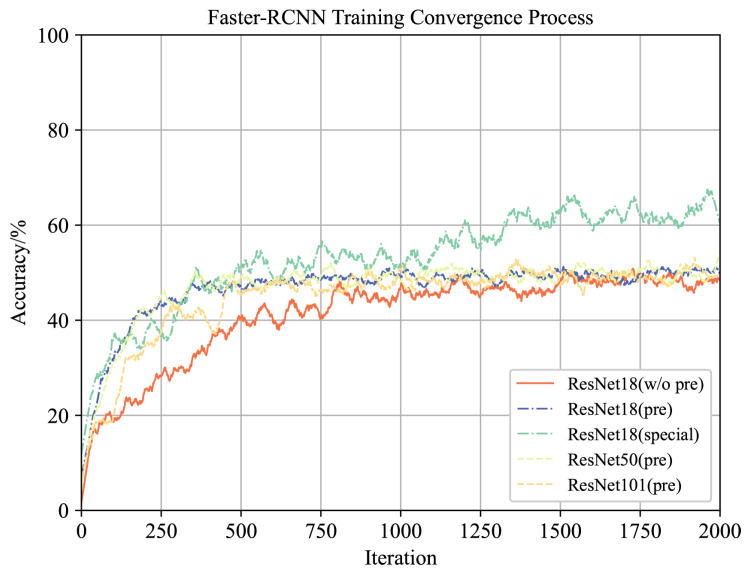
Training converegence process for Faster-RCNN.

**Figure A7:**
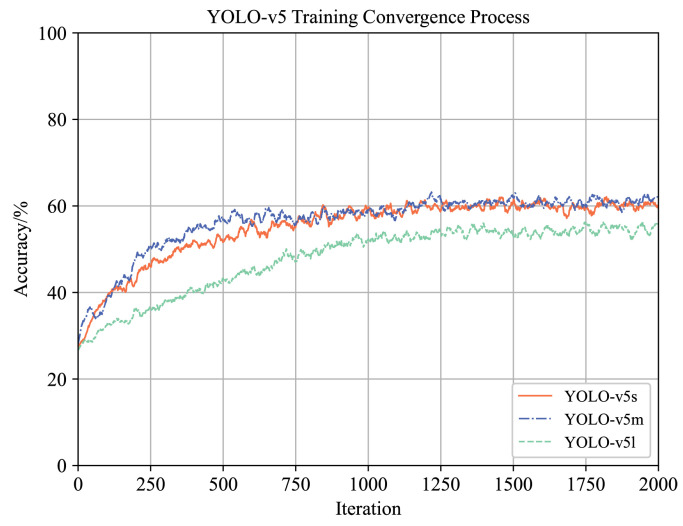
Training converegence process for YOLO.

**Figure A8:**
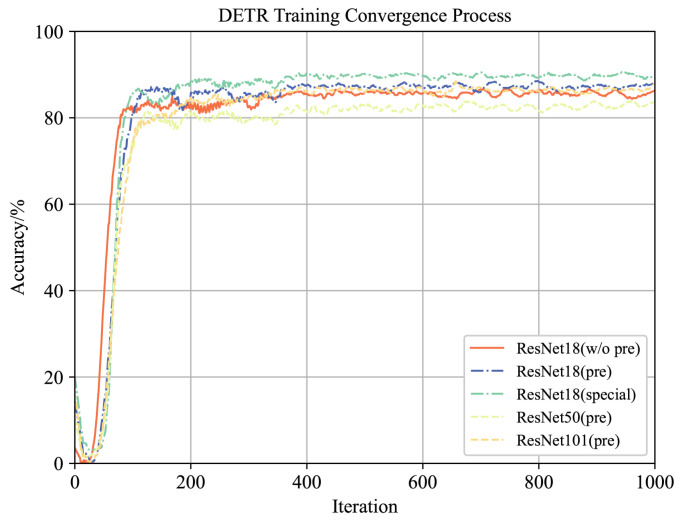
Training converegence process for DETR.

## Data Availability

The original contributions presented in this study are included in the article. Further inquiries can be directed to the corresponding author.

## Appendix A. Proof of Theorem 1

*Traditional shallow convolutional neural networks with 3×3 convolution kernels exhibit inherent proficiency in extracting small objects and non-edge features.*

**Proof.** Consider a CNN N with input tensor $I\in R^{H\times W\times C}$. The first convolutional layer $L_{1}$ employs 3×3 kernels $K^{\left(1\right)}\in R^{3\times 3\times C\times D}$ with stride 1 and padding 1, generating feature maps:

(A1) $$F_{d}^{\left(1\right)}\left(x,y\right)=\phi \left(\sum_{c=1}^{C}\sum_{i=-1}^{1}\sum_{j=-1}^{1}K_{i,j,c,d}^{\left(1\right)}\cdot I\left(x+i,y+j,c\right)+b_{d}\right)$$

where ϕ denotes the activation function. □

Part 1: Small Object Extraction Capability.

Define a small object O⊂I with spatial diameter ${∥O∥}_{\infty }\leq 3$ pixels (i.e., $\max_{(p,q)\in O}{∥p-q∥}_{\infty }\leq 3$). Let R(x,y) denote the 3×3 receptive field centered at (x,y).

**Lemma** **A1.**
*For any O satisfying ${∥O∥}_{\infty }\leq 3$, there exist coordinates $\left(x_{c},y_{c}\right)$ such that $R(x_{c},y_{c})\subseteq O$.*

**Proof.** By definition, O is contained within a 3×3 pixel region. Selecting $\left(x_{c},y_{c}\right)$ as the centroid of the minimal axis-aligned bounding box containing O satisfies the inclusion condition, with boundary handling guaranteed by the padding configuration. □

The activation strength for object O under kernel *d* is quantified as

(A2) $$A_{d}\left(O\right)=\max_{\begin{array}{c} (x,y):\\ R(x,y)\cap O\neq \emptyset \end{array}}\left|\sum_{c=1}^{C}{\langle K_{:,:,c,d}^{\left(1\right)},I_{R(x,y),c}\rangle }_{F}\right|$$

During gradient-based optimization,

(A3) $$\min_{K^{\left(1\right)}}L,\;\mathrm{where}\;L=-\sum_{O\in T}\log p\left(O|I\right);$$

the compact parameterization $\left(\dim(K^{\left(1\right)})=9CD\right)$ induces a spatial inductive bias. The limited receptive field constrains kernels to specialize in local patterns within their 3×3 support, naturally prioritizing small object detection.

Part 2: Non-edge Feature Extraction Capability. Define a non-edge feature N⊂I as a local pattern satisfying

(A4) $$G\left(N\right):=\max_{(x,y)\in N}{∥\nabla I\left(x,y\right)∥}_{2}\leq \tau$$

where τ is an edge detection threshold.

**Lemma** **A2.**
*For any N with diam(N)≤3, N is fully contained within some receptive field R(x,y).*

**Proof.** Spatial constraints ensure N resides within a 3×3 region. Selecting (x,y) as the center of this region guarantees N⊆R(x,y) under padding = 1. □

The representational capacity for N under kernel *d* is quantified as

(A5) $$R_{d}\left(N\right)=\max_{\left(x,y):N\subseteq R(x,y\right)}\left|\sum_{c=1}^{C}{\langle K_{:,:,c,d}^{\left(1\right)},I_{R(x,y),c}\rangle }_{F}\right|$$

During optimization,

(A6) $$\min_{K^{\left(1\right)}}L,\;L=-\sum_{N\in D}\log p\left(N|I\right),$$

the compact parameterization induces a structural prior.

**Lemma** **A3.**
*For any edge feature E with curvature $\kappa >\frac{\pi }{12}$, there exist distinct positions $\left(x,y\right)\neq \left(x^{\prime },y^{\prime }\right)$ satisfying*

(A7)
$$I_{R(x,y)}\approx I_{R(x^{\prime },y^{\prime })}\;\mathrm{but}\;E\cap R\left(x,y\right)≢E\cap R\left(x^{\prime },y^{\prime }\right)$$

**Proof.** Curvature $\kappa >\tan^{-1}\left(1/3\right)\approx 18.4^{\circ }$ causes significant geometric divergence of edge segments within 3×3 windows due to the discrete grid sampling. □

The constrained receptive field compels kernels to specialize in local pattern matching rather than global edge detection. By Lemma A3, edge features exhibit representation ambiguity while non-edge features satisfy

(A8) $$R_{d}\left(N\right)\propto {∥K_{:,:,:,d}^{\left(1\right)}∥}_{F}\cdot {∥N∥}_{F}$$

with minimal interference from edge components. Gradient descent therefore preferentially learns non-edge features within the $K_{\mathrm{non}\text{-}\mathrm{edge}}$ subspace.

## Appendix B. Typical Flight Formation Configuration and Parameters

Flight groups often adopt specific formation configurations to enhance reconnaissance and combat capabilities. These formations typically fall into six main types: Lead-Trail, Line Abreast, Swept, Wedge, Container, and Stinger, categorized based on the positional arrangement of wingmen in the formation, as illustrated in Figure A1.

The Lead-Trail formation typically comprises two to four aircraft, enabling efficient monitoring of wingmen and aerial situations to provide robust defensive support. The Line Abreast formation, consisting of two to four aircraft, offers extensive coverage of the attack zone, simplifying fire lane clearance and enabling effective attacks. Swept formations, also comprising two to four aircraft, provide flexibility for swift conversion into Lead-Trail or Line Abreast formations, making them ideal for low-altitude and adverse weather conditions. The Wedge formation, composed of three aircraft, facilitates tactical defense strategies, reduces the probability of hostile radar detection, and enables tactical deception. The Container formation, involving four aircraft, is commonly used for maintaining formation during cruising and executing specific air combat tactics such as the millstone. Lastly, the Stinger formation, comprising three aircraft, provides a clear forward BVR-fire channel and allows seamless support from the rear aircraft when needed.

The size and shape of formation configurations are adaptable and can be adjusted based on tactical considerations. Key parameters influencing the formation’s shape and size include depth, width, echelon, and slant in V-shape formations. Figure A2 illustrates the crucial parameters in the formation, while Table A1 details the specific value ranges of these key parameters as discussed in this paper.

**Table A1:** Hyperparameter setting of flight formation.

Parameters	Range
Aircraft spacing (km)	[5,15]
Oblique range of Swept (rad)	[0.314,1.256]∪[1.653,2.855]
Angel range of Wedge/Stinger (rad)	[0.523,1.047]
Horizontal and vertical noise (km)	[−1,1]
Course noise (rad)	[−0.2,0.2]
Formation numbers in one radar map	[1,20]

## Appendix C. Visualization of the Feature Extraction Layers

ResNet18, ResNet50, and ResNet101 [32] exhibit identical output image dimensions in their intermediate modules, with the primary difference being the number of convolutional layers across different modules. Theoretically, ResNet101 should at least match ResNet18 through an identity transformation, implying that ResNet101’s feature extraction capacity should exceed that of ResNet18. However, due to the uncontrollable nature of the network training, the actual feature extraction performance can vary between networks.

To investigate this, we applied the proposed image reconstruction method in a clustered scenario and passed the resulting images through the feature extraction networks to visualize key features within the network. Given the high number of image channels in the intermediate layers, we overlaid grayscale maps of each channel. The visualization results are presented in Figure A3.

We tested the impact of varying formation numbers in an image and observed consistent trends. As the image size was compressed from 256 × 256 to 28 × 28, the performance of the three networks remained similar, with larger networks like ResNet101 yielding slightly clearer compressed features. However, as compression continued to 14 × 14 and 7 × 7, particularly with more than five formations in the image, the results became increasingly difficult to interpret. Notably, in the 7 × 7 features of the final layer, deeper networks often generated compressed features with fewer pixels than the number of formations, which could explain the loss of certain features in sparse images. While shallow networks exhibit coarser feature granularity, they maintain consistency in the overall feature count.

## Appendix D. Pretraining Details

The first phase involves pre-training the E2I upsampling network using an autoencoder, as described in Section 3.3. Training occurs over 50 epochs, with 200 iterations per epoch and a batch size of 256. We set the initial learning rate to 0.001, with a decay rate of 0.1 every 10 epochs. SmoothL1Loss is employed alongside the Adam optimizer. Figure A4 showcases the convergence outcomes of the loss function during this training phase.

In the second step, a ResNet18 feature extraction network is trained using a supervised classification approach, freezing the pre-trained E2I upsampling network. This procedure aligns with the E2I-5 experiment in Section 4.2.2 and is not reiterated here. We use PCA [38] to visualize the compressed image features in a two-dimensional plane, as depicted in Figure A5.

The experimental results indicate that the feature extraction network, when specifically pre-trained, outperforms networks that lack pre-training or are pre-trained on ImageNet.

## Appendix E. Training Details for Faster-RCNN, YOLO, and DETR

The detailed experimental setups and optimized training parameters for the benchmark comparisons documented in Table 5 are sequentially described in the following discussion. Using the formation configuration data generation algorithm in Section 3.4, we simultaneously generate multiple flight formations within a single radar map to create a cluster configuration recognition scenario.

Faster-RCNN: We conduct a comparative analysis of Faster-RCNN using different backbone networks. Specifically, we examine ResNet18 (without pre-training, ImageNet pre-training, special pre-training), ResNet50 (ImageNet pre-training), and ResNet101 (ImageNet pre-training) to validate the effectiveness of our proposed Theorems 1 and 2, as well as the pre-training method. The initial learning rate is set at 0.001, decaying by 0.1 every 10 epochs. The training process consists of 20 epochs, with 100 iterations per epoch and a batch size of 6. We use the hybrid loss function from the original Faster-RCNN, depicted in Equation (A9).

(A9) $$L_{Faster\text{-}RCNN}=L_{cls}+L_{box\_reg}+L_{obj}+L_{rpn\_box\_reg}$$

where $L_{Faster\text{-}RCNN}$ denotes the total Faster-RCNN loss, $L_{cls}$ denotes the classification loss, $L_{box\_reg}$ denotes bounding box regression loss, $L_{obj}$ denotes the object loss, and $L_{rpn\_box\_reg}$ denotes the region proposal network bounding box regression loss. The convergence results of recognition accuracy are visually presented in Figure A6.

YOLO: We employ the widely recognized YOLO-v5 for end-to-end recognition, implementing the YOLO-v5 algorithm across three specifications: v5s, v5m, and v5l. The training initiates with a learning rate of 0.001, with a decay rate of 0.1 every 10 epochs. Training spans 20 epochs, each consisting of 100 iterations, with a batch size of 12. The classical mixing loss function of YOLO is utilized, as depicted in Equation (A10).

(A10) $$L_{YOLO}=aL_{box}+bL_{obj}+cL_{cls}$$

where $L_{YOLO}$ denotes the total YOLO loss, $L_{box}$ denotes the object bounding box loss, $L_{obj}$ denotes the object mask loss, and $L_{cls}$ denotes the classification loss. In the experiment, *a*, *b*, and *c* are set to 0.05, 1, and 0.5, respectively. The convergence outcomes regarding recognition accuracy across three distinct scales of YOLO-v5 networks are illustrated in Figure A7.

DETR: During DETR training, an initial learning rate of 0.001 is set, with a decay rate of 0.1 every 10 epochs. Training spans 20 epochs, each consisting of 50 iterations, with a batch size of 64. In this configuration, the hidden layer of the transformer is established at 256 units, employing a total of six layers for both the transformer encoder and decoder. Additionally, multi-head attention utilizes eight heads in its operation. We employ the mixed loss and Adam optimizer as proposed in the original DETR paper, utilizing the mixed loss and weight outlined in Equation (A11).

(A11) $$L_{DETR}=\alpha L_{ce}+\beta L_{bbox}+\lambda L_{giou}$$

where $L_{DETR}$ denotes the total DETR loss, $L_{ce}$ denotes the classification loss, $L_{bbox}$ denotes the bounding box regression loss, and $L_{giou}$ denotes the GIOU loss. In the experiment, α, β, and λ are set to 1, 5, and 2, respectively. Five distinct groups of independent experiments were conducted using various Backbone networks: ResNet18 (without pre-training, ImageNet pre-training, special pre-training), ResNet50 (ImageNet pre-training), and ResNet101 (ImageNet pre-training). The convergence results of recognition accuracy are visually presented in Figure A8.
